# Diagnostic Utility of Antibodies to Extractable Nuclear Antigens in the Absence of Positive Antinuclear Antibodies

**DOI:** 10.7759/cureus.93419

**Published:** 2025-09-28

**Authors:** Jason Huang, Bassel B Dargham, Michael Luggen

**Affiliations:** 1 Internal Medicine, University of Cincinnati Medical Center, Cincinnati, USA; 2 Rheumatology, University of Cincinnati Medical Center, Cincinnati, USA

**Keywords:** ana, autoantibodies, autoimmune diseases, ena, practice guidelines, rheumatology

## Abstract

Objective: The antinuclear antibody (ANA) test by indirect immunofluorescence (IFA) is highly sensitive for systemic autoimmune rheumatic diseases (SARD), however, its specificity is low, necessitating additional testing for antibodies to extractable nuclear antigens (ENA). The American College of Rheumatology recommends screening with the ANA by IFA but advises against testing further if the ANA is negative. This is based primarily upon consensus opinion and a limited number of studies. This study further investigates the utility of an isolated positive ENA in patients with negative ANA followed over time and evaluated by a rheumatologist, the first such study to do so.

Methods: All patients aged ≥18 years seen at the University of Cincinnati Medical Center who tested negative for ANA and positive for ENA within a 30-day window between April 2015 and April 2024 and were evaluated by a rheumatologist on at least one occasion constituted the study population. Clinical diagnoses were established by board-certified rheumatologists and independently verified through electronic medical record review by a second rheumatologist.

Results: Among 4,462 patients undergoing ANA testing with subsequent rheumatology evaluation, 1,594 had concurrent ENA testing. Of these, 72 patients (4.5%) exhibited ENA positivity despite negative ANA results. Only four of the 72 ANA-negative, ENA-positive patients (5.6%) received a new diagnosis of a SARD.

Conclusions: The utility of testing for ENA when the ANA by IFA is negative is low. The vast majority of positive tests are false positives that lead to additional testing at additional costs.

## Introduction

Antinuclear antibody (ANA) testing remains a cornerstone in the evaluation of systemic autoimmune rheumatic diseases (SARDs), demonstrating a sensitivity exceeding 95% for the clinical diagnosis of systemic lupus erythematosus (SLE), approximately 90% for systemic sclerosis and approaching 100% for mixed connective tissue disease (MCTD) [1,2]. While a positive ANA result often guides subsequent diagnostic workup, its specificity remains low with a positive predictive value of just 12.8% [3], necessitating further testing for more specific extractable nuclear antigen (ENA) antibodies. ENA testing generally assesses antibodies to anti-Smith (Sm), anti-ribonucleoprotein (RNP), anti-Sjogren's syndrome A and B (SS-A and SS-B, also referred to as Ro and La respectively), anti-histidyl-tRNA synthetase (Jo-1), anti-topoisomerase I (Scl70), and anti-DNA-histone complex (chromatin). Antibodies to double-stranded DNA (dsDNA) are not commonly considered an ENA antibody but automated test systems routinely include this in the ENA profile and it will be included as such here.

Antibodies to ENA are more specific for a particular SARD but less sensitive. The specificity of anti-dsDNA for lupus, for example, is 89-99% depending on test system and concentration of antibody. The specificity of anti-Sm for SLE is even higher at 98-99%. Antibodies to Scl70/topisomerase I have a 90% specificity for systemic sclerosis and antibodies to SS-A have a specificity of 98% for primary Sjogren's [4].

The American College of Rheumatology (ACR) recommends screening for SARD with an ANA by indirect immunofluorescence (IFA). If positive and if a clinical suspicion, the ACR recommends further testing with an ENA. However, if ANA by IFA is negative, ENA testing is recommended against [5]. Nonetheless, ENA testing in ANA-negative patients continues to be performed. Occasionally, the ENA is positive under such circumstances. The significance of such a result is not completely clear. A limited number of studies have investigated the diagnostic yield of isolated ENA positivity in ANA-negative individuals [6-10]. These investigations have all been retrospective and cross-sectional, primarily focused on laboratory test utilization. Clinical data have generally been limited, and none of the studies have required specialist evaluation. Our study addresses these design limitations by requiring each included case be assessed longitudinally by two board-certified rheumatologists.

## Materials and methods

This retrospective study was conducted at the University of Cincinnati Medical Center, a tertiary medical center in Cincinnati, Ohio, and included all patients aged ≥18 years who tested negative for ANA and positive for ENA antibodies within a 30-day interval between April 2015 and April 2024. Inclusion criteria required at least one rheumatology evaluation during the study period. The study protocol was reviewed and approved by the University of Cincinnati Institutional Review Board (approval 00000180).

ANA testing was processed by LabCorp (Laboratory Corporation of America Holdings), Dublin Regional Laboratory (Dublin, OH, USA). Prior to June 18, 2016, ANA testing employed the ANA PolyTiter immunofluorescence system (Polymedco, Inc., Cortland Manor, NY, USA) along with the ANAFLUOR HEp-2 reagent kit (DiaSorin, Stillwater, MN, USA). After this date, ANA was analyzed using a fully automated IIF platform (Sprinter XL, EUROPattern Suite, Euroimmun AG, Lübeck, Germany) utilizing IFA 40: HEp-20-10 cells. Studies have demonstrated 95.6% concordance between the two methodologies for determining ANA positivity [11]. A titer of ≥1:80 was used as the threshold for a positive result.

ENA antibody testing was performed throughout the entire study period by LabCorp using the BioPlex 2200 system (Bio-Rad, Hercules, CA, USA), a multiplex immunoassay platform detecting anti-dsDNA, anti-RNP, anti-Sm, anti-SSA, anti-SSB, anti-Scl-70, anti-Jo-1, and anti-chromatin antibodies. Assay interpretation followed manufacturer-supplied standards, and results were categorized as positive based on LabCorp’s reference criteria.

Clinical diagnoses were assigned by a board-certified rheumatologist and independently verified through electronic medical record review by a second rheumatologist. Most patients, including ANA-negative, ENA-positive patients, were seen on multiple occasions, providing adequate follow-up to capture evolving clinical features. Demographic and clinical data were extracted from the electronic medical record. ANA and ENA testing were not linked at our institution; ordering decisions were made at the discretion of the treating physician.

## Results

**Figure 1:**
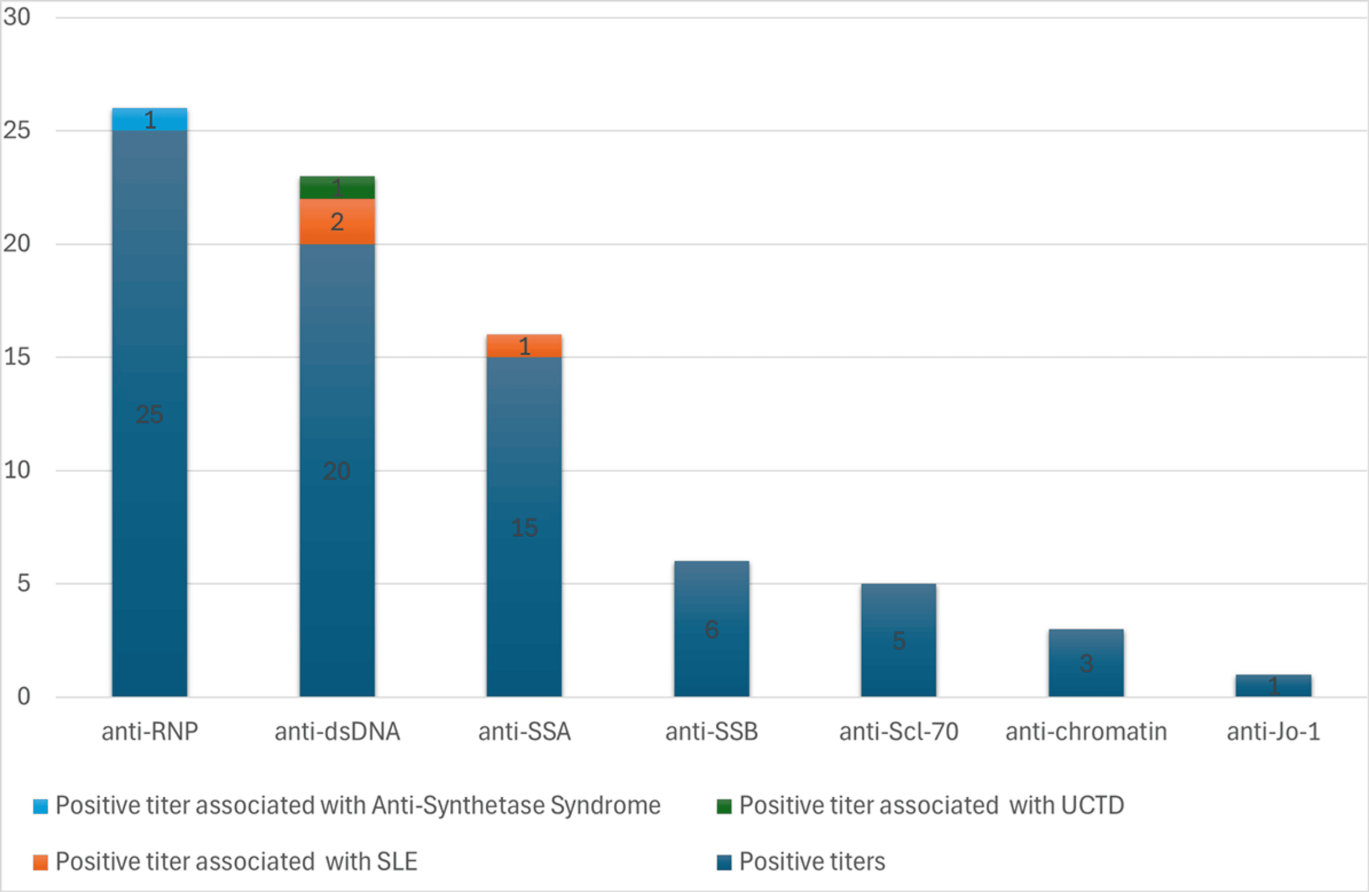
Bar graph depicting the distribution of positive extractable nuclear antigen (ENA) sub-serologies in setting of negative antinuclear antibody (ANA) with associated systemic rheumatic diseases SLE: systemic lupus erythematosus, UCTD: undifferentiated connective tissue disease.

Among 4,462 patients undergoing ANA testing with subsequent rheumatology evaluation, 1,594 had concurrent ENA testing. Of these, 72 patients exhibited ENA positivity despite negative ANA results. The distribution of positive ENA antibodies was as follows: 26 anti-RNP, 23 anti-dsDNA, 16 anti-SSA, six anti-SSB, five anti-Scl-70, three anti-chromatin, two anti-Sm and one anti-Jo-1, with an occasional patient having more than one positive ENA (Figure 1).

Of our 72 patients, 62 had more than one visit to a rheumatologist and were followed for a median duration of 14.2 mos (6.1 and 13.9 for 25th and 75th percentile respectively).

**Figure 2:**
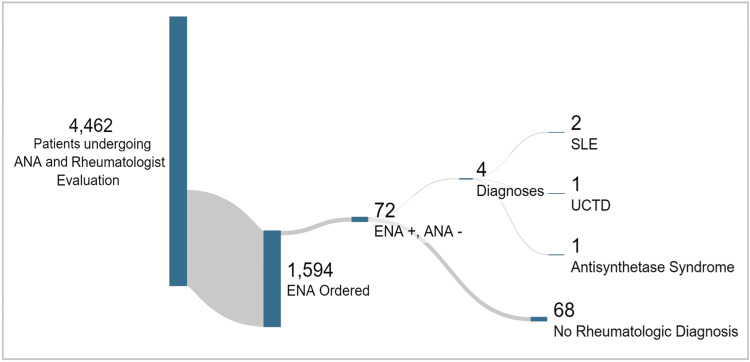
Distribution of patients studied ANA: antinuclear antibody, ENA: extractable nuclear antigens, SLE: systemic lupus erythematosus, UCTD: undifferentiated connective tissue disease.

Only four of the 72 ANA-negative, ENA-positive patients (5.6%) received new autoimmune disease diagnoses: two with SLE, one with undifferentiated connective tissue disease (UCTD), and one with anti-synthetase syndrome. The individual with diagnosed anti-synthetase syndrome was notably anti-PL-7 positive on supplemental testing. The remaining 68 (94.5%) either had no SARD or a pre-established diagnosis (Figure 2).

## Discussion

Our findings underscore the limited diagnostic utility of ENA testing in ANA-negative patients. Only 5.6% of such patients in our study were diagnosed with a new SARD by a rheumatologist after a median period of observation of 14.2 months. These results are similar to those of Yeo et al., who found a diagnostic yield of 6.1% [6]. Comparable results were also found by Davis et al. [8] and Homburger [9] while Ethington et al. [7] found an even lower positive predictive value. However, similar to the study by Yeo et al., these prior analyses predominantly focused on laboratory data and readily available, cross-sectional clinical information from a number of different types of physicians [6-9]. While such approaches offer valuable insights into test performance characteristics, they have certain limitations. Given that the diagnosis of systemic rheumatic diseases typically relies on a combination of carefully evaluated clinical features as well as critical interpretation of serologic findings, evaluation by a rheumatologist is essential. Longitudinal follow-up is also critical, as many rheumatic diseases evolve over time. Our goal was to confirm previous observations with more rigorous methods, which we believe we have done.

In terms of clinical diagnoses, two patients (2.8% of ANA-negative, ENA-positive patients) were diagnosed with ANA-negative SLE based upon their compelling constellation of clinical features, one of 72 patients (1.4%) met criteria for anti-synthetase syndrome and one was felt to have a UCTD. These numbers are small and difficult to compare with previous studies, but SLE was among the most common clinical diagnosis found by other investigators as well.

In our cohort, certain ENA specificities were more frequently observed among ANA-negative patients, with anti-RNP and anti-dsDNA being the most common. Shovman et al. also found these to be the most common false positives among 510 randomly selected blood donors [12].

At our institution, the cost of ANA testing with reflex to pattern and titer is approximately $105 USD, whereas antibodies to an ENA panel is substantially higher, with a cost of approximately $1,170.00 USD. While exact costs may vary depending on geographic location and the extent of the ENA testing panel, the cost differentials are likely comparable. In our patient population, the laboratory cost to detect a single case of a SARD was over $28,000 given the false positive rate. This does not include cost for additional testing which is often done and the cost of consultation. False positive results also may result in loss of time from work or decreased productivity and may also contribute to patient anxiety. The findings of our study align with current ACR guidelines, which recommend limiting ENA testing to cases with confirmed ANA positivity and a clinical suspicion of a SARD and further support the Choosing Wisely initiative aimed at enhancing the cost-effectiveness of healthcare resource utilization [5].

Limitations

The primary limitation of our study is sample size. Although our initial cohort comprised nearly 4,500 patients, only 72 met the criteria for ANA-negative, ENA-positive status who also had a rheumatologic evaluation. While this finding underscores the rarity of this serological profile, it limits the precision of the estimate and the statistical power for subgroup analyses. Another potentially important limitation is the duration of follow-up. Some SARDs present acutely, while others may evolve gradually over several years [13]. It is therefore possible that a subset of patients with slowly evolving disease was not captured. However, by including the category of UCTD, we aimed to encompass all patients with clinically suspicious features, even in the absence of a definitive diagnosis. The number of patients who were evaluated, did not meet even the UCTD definition, and those who subsequently developed a definite SARD is likely to be very small.

## Conclusions

The detection of ENA positivity in ANA-negative patients has a very low positive predictive value for systemic autoimmune rheumatic diseases, reinforcing current guidelines that recommend ENA testing only following a positive ANA result. Important healthcare cost savings could be realized by following these guidelines.
